# Prevalence of Peri-Implant Diseases in a Private Practice and Potential Risk Indicators 

**DOI:** 10.3290/j.ohpd.c_1805

**Published:** 2025-01-22

**Authors:** Codruta Elena Ciurescu, Lorena Dima, Anca Gheorghiu, Vlad Alexandru Ciurescu, Dana Gabriela Festila, Marius Alexandru Moga, Stefan Vesa, Raluca Cosgarea

**Affiliations:** a Codruta Elena Ciurescu PhD Student, Transylvania University of Brasov, Faculty of Medicine, Department of Fundamental Disciplines and Clinical Prevention, Brasov, Romania; Transylvania University of Brasov, Faculty of Medicine, Department of Fundamental Disciplines and Clinical Prevention, Brasov, Romania; Oral surgeon, private periodontal practice, Brasov, Romania. Conceptualisation, data curation, wrote original draft.; b Lorena Dima Specialist in Clinical Pharmacology, Transylvania University of Brasov, Faculty of Medicine, Department of Fundamental Disciplines and Clinical Prevention, Brasov, Romania; Transylvania University of Brasov, Faculty of Medicine, Department of Fundamental Disciplines and Clinical Prevention, Brasov, Romania. Periodontal private practice, Brasov, Romania. Investigation, resources, data curation, conceptualisation, validation, reviewed and edited the manuscript, visualisation, supervision.; c Anca Gheorghiu Periodontist, Iuliu Hatieganu University of Medicine and Pharmacy Cluj-Napoca, Faculty of Dental Medicine, Cluj-Napoca, Romania. Software, resources, data curation, wrote original draft.; d Vlad Alexandru Ciurescu Medical Undergraduate Student, Department of Radiology and Maxillofacial Surgery Department, Faculty of Dental Medicine, Iuliu Hatieganu University, Cluj Napoca, Romania. Software, resources, wrote original draft.; e; f Dana Gabriela Festila Orthodontist, Transylvania University of Brasov, Faculty of Medicine, Department of Medical and Surgical Specialties, Brasov, Romania. Validation, formal analysis, visualisation, supervision.; g Marius Alexandru Moga Medical Doctor, Department of Pharmacology, Toxicology and Clinical Pharmacology, “Iuliu Hatieganu” University of Medicine and Pharmacy, Cluj-Napoca, Romania. Methodological planning, checked data entry.; h Stefan Vesa Medical Doctor, Statistician, Department of Periodontology, Operative and Preventive Dentistry, University of Bonn, Bonn, Germany. Performed the statistical analysis.; i Raluca Cosgarea Periodontist, Assistant Professor and Prodean, Department of Periodontology, Operative and Preventive Dentistry, University of Bonn, Bonn, Germany; Periodontist, Assistant Professor, Department of Periodontology and Peri-implant Diseases, Philips University Marburg, Marburg, Germany; Periodontist, Assistant Professor, Department of Science, University Iuliu-Hatieganu, Cluj-Napoca, Romania. Rewriting and reanalysis.

**Keywords:** inflammation, infection, peri-implant disease, peri-implant mucositis, peri-implantitis, prevalence

## Abstract

**Purpose:**

The purpose of this study was to evaluate the occurrence of peri-implant diseases and their potential risk indicators in a private practice setting.

**Materials and Methods:**

This cross-sectional study evaluated data from 390 subjects (mean age 55.8 ± 11.6 years) with implant-supported prosthetic reconstructions, who were enrolled in a maintenance program for 6.25 ± 3.36 years. Clinical evaluation included peri-implant probing pocket depth (PPD), bleeding on probing (BOP) and full-mouth plaque scores (FMPS). Radiographic evaluation was performed using retro-alveolar radiographs for each implant. Further, smoking habits, history of periodontitis, or tooth loss due to periodontal disease, presence/absence of keratinized mucosa ≥ 2 mm and the quality of the prosthetic restoration were also assessed. The prevalence of the peri-implant disease (at the subject/implant level) was determined and various potential risk indicators were evaluated by multi-level logistic regression analysis.

**Results:**

The prevalence of peri-implant diseases was 37.7% and 23.3% at the subject and implant level, respectively. 14.3% of the subjects were diagnosed with peri-implant mucositis and 8.9% were diagnosed with advanced peri-implantitis (PI). PI was statistically significantly associated with poor (FMPS > 0.45, p < 0.001) or moderate oral hygiene (FMPS: 0.3–0.45, p < 0.001), a history of periodontitis (p < 0.001), lack of keratinized tissue ≥2 mm (p < 0.001) or implant function time > 5 years (p < 0.001).

**Conclusion:**

In a private practice setting, a prevalence of peri-implant diseases of 37.7%/ 23.3% (subject/implant level) was found. Poor oral hygiene, history of periodontitis, a keratinized mucosa < 2 mm and a time in function ≥ 5 years have been associated with the occurrence of peri-implant diseases.

PERIODONTOLOGY

Today, dental implants are employed globally to restore edentulous dental arches. High long-term survival rates of dental implants have been reported in several studies: 98.8%,^2^ 97.96%,8 96.9%,^11^ 95.1%,^12^ and 95%,^18^ making this treatment option a very attractive alternative to conventional prosthetic options for replacing missing teeth. With dental implants, patients’ quality of life can be improved significantly by restoration of function, esthetics and phonetics. However, despite the reported high survival rates of implants, an increasing number of patients is experiencing peri-implant diseases.

High survival rates have been reported both for systemically healthy (cumulative survival rates of 83.8% after 25 years, 96.1% after 10 years) as well as for medically compromised patients (cumulative survival rate after 20 years 90.8%).^11^ After 20 years in function, an implant survival rate of 93% in periodontally treated or periodontally healthy subjects has been documented.^32^ Despite the high survival rates, implant failures may still occur.^12,17,26^ In the last decade, evidence on the presence of peri-implant diseases affecting both soft and hard tissues that may eventually lead to implant loss has substantially increased. These are seen as biological complications related to inflammatory conditions of the surrounding soft tissues and bone, which are induced by bacterial biofilm and are distinguished as peri-implant mucositis and peri-implantitis.^7,10,21^ Consequently, it seems essential to determine not only the impact of peri-implant diseases upon the survival rates, but also to assess their risk factors/indicators in order to prevent the occurrence of these diseases as much as possible.

When first described in 1987 by Mombelli et al,^27^ peri-implantitis (PI) was considered an infectious disease with many similarities to periodontitis. Based on several clinical symptoms and etiological factors that have been described since then, a plethora of definitions have arisen in the past decades. PI was mainly defined as an inflammatory reaction of the peri-implant mucosa with marginal bone loss, while peri-implant mucositis involved only soft-tissues inflammation with no clearly defined values for the amount of tissue loss.^25,19^ Discrepancies in case definitions resulted in a wide variation in the reported prevalence values for peri-implant diseases.^4,41^ The lack of clear clinical parameters in these definitions has led to difficulties in setting treatment guidelines. Based on clear definitions for incidence (the number of new cases of a specific disease occurring during a certain period) and prevalence (the number of cases of a disease in existence at a certain time point),^20^ longitudinal studies have been proposed for determining the incidence, but cross-sectional studies for the prevalence of peri-implant diseases.^19^

Fortunately, in November 2017 at the World Workshop on Periodontolgy (WWP), the European Federation of Periodontology (EFP) and the American Academy of Periodontology (AAP) set clear definitions with clinical cut-off points for peri-implant diseases both for the day-to-day clinical practice as well as for epidemiological studies.^40^ Moreover, in 2020, similar to the periodontal risk assessment,^23^ an assessment tool for the risk of developing peri-implant diseases has been proposed based on eight risk vectors: assessment of history of periodontitis, percentage of sites with bleeding on probing (BOP), periodontitis susceptibility (based on the current classification of periodontal and peri-implant diseases),^3,43^ frequency/compliance with supportive periodontal therapy, number of teeth/implants with probing pocket depths (PPD), ratio of bone loss to age, distance from the restorative margin of the implant prosthetic reconstruction to the marginal bone crest, and prosthetics-related factors.^14^ Some of these factors, including insufficient periodontal treatment as well as untimely diagnosis and management of mucositis or non-compliance with supportive peri-implant care, are well-documented risk factors. However, there is poor evidence for improper implant position/wrong implant placement, incorrect prosthetic suprastructures, poor postoperative care and missing assessment and appropriate treatment of soft-tissue defects as risk factors.^6,33,39^ In light of their bacterial etiology, documented risk factors, and the aggressive, uncontrollable nature of peri-implant diseases, thorough routine clinical assessment including regular probing and accurate radiographic assessment are strongly recommended for correct diagnosis of peri-implant diseases.^1,6,28,30,37^ An early diagnosis, preventive measures, and adherence to individually tailored peri-implant support play a major role in the prevention and management of peri-implant diseases.^1,30^

Considering the various prevalence data on peri-implant diseases, the aim of the current study was to evaluate the occurrence of peri-implant diseases (i.e., peri-implant mucositis and peri-implantitis) in a private dental setting based on the current classification. Additionally, we analysed the presence of the major risk factors for peri-implant diseases, as defined by the IDRA tool.

## MATERIALS AND METHODS

This was a cross-sectional study that evaluated patient data with implant-supported fixed restorations from a private dental clinic (Krondent; Brasov, Romania). The study obtained the approval of the local Ethics Committee of the Transylvania University of Brasov, no. 30/06) and all patients signed an informed consent to participate in the study before it began.

### Inclusion and Exclusion Criteria

All included patients had been enrolled in a maintenance program at the private dental clinic for at least one year from the finalisation of the implant prosthetic restoration.

General medical history as well as smoking habits (smoking ≥ 10 cigarettes/day), history of tooth loss caused by periodontal disease, and history of periodontitis were recorded. Considering that smoking ≥ 10 cigarettes/day modifies the grade for periodontitis,^29,43^ the present study considered only those smoking ≥ 10 cigarettes/day as smokers. Information on history of periodontitis was extracted from the patients’ records, since all patients had been patients at the clinic. Medical history including diseases and medication with an impact on the periodontium – e.g., diabetes, stress, obesity or depression – were recorded.

Excluded from the study were patients aged < 18 years, those with an implant time in function < 12 months, those who had taken antibiotics in the previous 3 months, had a prosthetic restoration with impaired access for clinical measurements, or were unwilling to participate in the study.

### Case Definitions

Peri-implant mucositis and PI were defined according to the World Workshop on Classification of Periodontal and Peri-implant Diseases and Conditions 2017, Chicago.^15,40^ Peri-implant mucositis was diagnosed considering presence of local signs of inflammation around the implant (redness, swelling, bleeding on local probing or suppuration on probing) and absence of peri-implant bone loss as assessed on retroalveolar radiographs taken at baseline (6–12 months after function of the prosthetic suprastructure).

PI was diagnosed considering presence of peri-implant signs of inflammation (bleeding or suppuration on probing), radiographic evidence of peri-implant bone loss after the initial (post-prosthetic) bone remodeling period and increased PPD compared to the values obtained after prosthetic reconstruction. When initial examination data were not present, 3 mm peri-implant bone loss from the implant shoulder, PPD ≥ 6 mm and bleeding on probing (BOP) were considered as parameters for diagnosis.

### Assessment of Clinical and Radiographical Parameters

All measurements were performed by a previously calibrated examiner (AG). PPD and BOP were measured to the nearest millimeter at 6 sites (mesio-buccal, buccal, disto-buccal, mesio-oral, oral, disto-oral) at teeth and implants using a mm-scaled periodontal probe (PCPUNC Probe 15 mm, Hu Friedy; Chicago, IL, USA). Care was taken to keep the probe vertical as much as possible. BOP was dichotomously (1: bleeding, 0: no bleeding) noted at each probing site 15 s after retracting the probe. Full-mouth plaque scores (FMPS) were assessed using Approximal Plaque Index score.^24^

The amount of peri-implant keratinized tissue was measured to the nearest millimeter using a mm-scaled periodontal probe and sites were scored as following: 1: keratinized tissue ≥ 2 mm; 0: keratinized tissue < 2 mm at least on one side of the implant.

Bone loss was assessed on retroalveolar radiographs at the mesial and distal aspect of each implant. Bone loss was measured from the most apical implant-bone level contact to the implant shoulder. Intraoral radiographs were taken using film holders to ensure paralleling technique and reduce distortion of the image. The radiographs were analysed using ClinicView Software (Instrumentarium Dental Imaging Software; Milwaukee, WI, USA), which provides measurement tools that are calibrated to the size of the radiograph.

Additionally, the quality of the prosthetic restoration was assessed clinically and radiographically and coded as follows: code 1: 2-mm distance from the implant shoulder to the restoration margins and optimal abutment-restoration fit; code 0: the previously mentioned criteria were not met so the restoration was considered “ill-fitting”.

Potential risk indicators were considered history of periodontitis, poor plaque control and lack of adherence to the maintenance program (good adherence: 1–2 visits per year). Factors possibly influencing the host response were also recorded: genetic factors, smoking habits, diabetes, stress, obesity, and depression. The consumption of antibiotics and other medications were also considered. Additionally, data regarding implant insertion and loading protocols, as well as the history of periodontal treatment, were recorded.

### Statistical Analysis

Statistical analysis was performed using MedCalc Statistical Software version 20 (MedCalc Software; Ostend, Belgium; https://www.medcalc.org). Quantitative variables were tested for normality of distribution using the Shapiro-Wilk test and were expressed as median and 25–75 percentiles. Qualitative variables were characterised by frequency and percentage. Comparisons between groups regarding the quantitative variables were performed using the Mann-Whitney test. Comparisons between groups regarding the qualitative variables were performed using the chi-squared test. Variables that achieved statistical significance in the univariate analysis, were introduced in a multivariate logistic regression. A p-value < 0.05 was considered statistically significant.

In order to assess the impact of the risk indicators on the occurrence of peri-implant diseases, multilevel logistic regression models were applied.

## RESULTS

The demographic data of the study group are presented in Table 1.

441 subjects who have been enrolled in a maintenance program in a private dental clinic (Krondent, Brasov, Romania) were screened to be included in the present study. Of these, 390 agreed and were finally considered for the analysis. The following reasons for exclusion were: two subjects refused to sign the consent, 24 subjects had prosthetic restorations with a time in function <12 months, 18 had taken antibiotics in the previous 3 months (n = 18), three subjects had an inadequate prosthetic restoration that impaired taking proper clinical measurements.

Thus, 390 patients (median age: 53 years [range 45 to 62 years]; female patients: n = 203 /52.05%; smokers: n = 99/ 25.4%)^30^ with 1639 implants (nonlinear value distribution, median number/patient: 3 [2;6]) were included in the study. The majority of the patients were systemically healthy (89.7%) with only 10.3% stating systemic diseases, including diabetes, depression, obesity or high blood pressure. Similarly, most patients had no history of treated periodontitis (82.3%) and only a low percentage of patients showed a poor oral hygiene (16.9%) (Table 1). Implants were mostly located in the lateral area (43% in the maxilla, 41% in the mandible) and only 16% were located in the frontal area (10% maxilla, 6% mandible).

Peri-implant diseases occurred in 23.3% of the implants, of which 14.3% were diagnosed with peri-implant mucositis and 8.9% PI. Eleven implants in nine subjects were lost in the follow-up after loading. Of these, one implant in one subject had been lost within the first 12 months after loading, whereas the others had been explanted for the following reasons after a function time ≥ 5 years: 6 implants due to wrong implant position, 2 implants due to implant/screw fracture, 2 implants were explanted with unknown indication by other colleagues.

Of the patients who were diagnosed with peri-implant diseases, good hygiene parameters were recorded only in 18 patients (12.2%), whereas good oral hygiene was observed in 57.2% of the patients with healthy implants. Vice versa, poor oral hygiene was statistically significantly more observed in patients with peri-implant diseases (40.8% vs 2.5%).

The univariate logistic regression analysis used to determine independent association between the occurrence of peri-implant diseases and various parameters indicated a statistically significant relation to the number of implants per patient, time in function, history of periodontitis, oral hygiene, smoking ≥ 10 cigarettes/day, medical history, inadequate prosthetic restoration and keratinized tissue width (Table 2).

When considering all the previously determined, statistically significant associations from Table 2, the multivariate regression analysis confirmed that a history of periodontitis, a time in function ≥ 5 years, poor oral hygiene and a keratinized tissue width < 2 mm has a statistically significant impact on the occurrence of peri-implant diseases (Table 3). The strongest statistically significant risk associations seem to be poor oral hygiene (OR 67.884, CI [22.28; 206.827]), followed by history of periodontitis (OR 14.825, CI [5.790; 37.961]) and a narrow width of keratinized tissue (< 2 mm) with an OR of 13.914 (CI [4.965; 38.995], Table 3).

Smoking ≥ 10 cigarettes a day, inadequate prosthetic reconstruction or medical history did not seem to have any statistically significant impact on the occurrence of peri-implant diseases (Table 3).

Table 4 shows the distribution of peri-implant diseases at the patient- and implant-level, based on the time in function (< 5 years and ≥ 5 years). 10% of the subjects were diagnosed with both peri-implant mucositis as well as PI, of which the majority had implants ≥5 years in function. A similar pattern was observed when analysing the patients with implants diagnosed only with mucositis or PI: a higher number of subjects/implants showed peri-implant diseases when the implants were in function for ≥ 5.

## DISCUSSION

Our study addressed the prevalence of peri-implant diseases in a private practice setting. Additionally, some potential risk indicators and their association with peri-implant diseases were assessed. Data from 390 patients, all treated and enrolled in a maintenance program at the same practitioner (CC) from a private dental clinic were considered for the study analyses.

Our findings show that 23.3% of the implants and 37.7% of the patients were diagnosed with peri-implant diseases. At the implant level, PI was present in 8.9% implants and peri-implant mucositis in 14.3%. Additionally, the number of implants/patient, time in function, history of periodontitis, oral hygiene, smoking ≥10 cigarettes/day, medical history, inadequate prosthetic restoration and keratinized tissue width were statistically significantly associated with the occurrence of peri-implant diseases.

These results are comparable to those reported in other studies and systematic reviews. In 2012, Mombelli et al^26^ reported a prevalence of 10% at the implant level and 20% at the patient level 5–10 years after implant placement. Daubert et al^7^ reported a 48% prevalence for peri-implant mucositis (95% CI: 39–59) at the patient level, and 26% for PI (95% CI: 18–37). After a mean follow-up of 10 years, 33% of implants were diagnosed with peri-implant mucositis (95% CI: 26–43) and 16% with PI (95% CI: 11–23). Higher numbers were reported by Derks and Tomasi,^10^ who found PI in 22% of the patients (95% CI: 14–30) and peri-implant mucositis in 43% (95% CI:19–65). Kordbacheh Changi et al^21^ evaluated the prevalence, incidence and possible risk factors in 2018 based on an electronic database from dental schools. PI occurred in 34% of the patients and 21% of the implants, with implants being in function over 2 years.^21^ According to another review, the prevalence of PI ranges between 0% and 40%.^11^ From a private practice, Roccuzzo et al^35^ reported an occurrence of 67.9% for peri-implant mucositis and 10.6% for peri-implantitis after 10 years. After 20 years, the prevalence for mucositis decreased to 47.5%, whereas that of peri-implantitis increased 3-fold to 33.3%.^35^ The difficulties in comparing the results were due to the broad case definition and heterogeneous thresholds used for defining for peri-implant bone loss.

As previously stated in other publications,^5,11,38^ the prevalence of peri-implant diseases varies greatly among the clinical studies described in the literature due to the various case definitions of the peri-implant infections and study design, but also due to the different methods of reporting.

### Risk Factors

As mentioned in the recently published peri-implantitis guidelines, peri-implant biofilm accumulation is recognised as the primary etiological factor.^16^ Additionally, a history of periodontitis, poor plaque control, and irregular supportive peri-implant care (SPIC) were recognised as important risk factors/indicators.^34,39^ Another recent study reported a survival rate of 93% after 20 years in function in periodontally compromised and healthy patients.^32^ Statistically significantly more periodontally compromised patients experienced suppuration and biological complications that required cumulative interceptive supportive therapy over this period.^32^

Smoking, diabetes, presence of submucosal cement, implant position with limited access for oral hygiene and maintenance were unclear in their role as risk factors for peri-implant diseases. Moreover, the EFP proposed further risk factors including absence of peri-implant keratinized mucosa, occlusal overload, bone compression necrosis, overheating during implant insertion, micromotion, biocorrosion or presence of titanium particles within the peri-implant tissues.^16^ Consequently, the present study analysed the association between the occurrence of peri-implant diseases and a history of periodontitis, full-mouth oral hygiene, smoking, systemic medical diseases, quality of the prosthetic reconstruction, gender, and width of the peri-implant keratinized mucosa. In the univariate regression analysis, a history of periodontitis showed the highest risk for peri-implant diseases with an OR of 15.6 (7.62–31.8) followed by smoking with an OR of 3.21 (2–5.14). The other investigated risk indicators showed only a slightly statistically significant association. Moreover, in the multivariate analysis, poor oral hygiene had the highest impact on the occurrence of peri-implant diseases (OR: 67.8; CI: 22.28–206.82). A history of periodontitis and the presence of peri-implant keratinized tissue width < 2 mm were also statistically significantly associated with peri-implant diseases (OR: 14; CI: 5.7–37.96 and 13.91; CI: 4.96–38.99). Additional stastisticall significant risk indicators were implant time in function ≥5 years and moderate oral hygiene. Interestingly, smoking over 10 cigarettes/day lost its statistical significance in the multivariate analysis (p = 0.6) (Table 3). It is important to emphasise that the present results confirm the recognised and proposed risk factors/indicators of the EFP.^16^ The evaluated risk-factors in our analysis are also recognised predictive vectors for risk assessment for developing peri-implantitis (Implant Disease Risk Assessment, IDRA).^14^

The present results corroborate those of other studies. Based on a multivariate logistic regression analysis, Kordbacheh Changi et al^21^ reported a statistically significant association between occurrence of PI, the fit of fixed prosthesis (OR = 5.9%, CI: 1.6-21-1), a cement–retained prosthesis (OR +3.6, CI: 2.1–9.5) and a history of periodontitis (OR = 3.6, CI:1.7–7.6). In the present multivariate analysis, the highest association was related to poor oral hygiene (OR 67.5) followed by a history of periodontitis and lack of keratinized tissue (Table 3). The statistically significant correlation between peri-implant diseases and poor oral hygiene has also been reported by other authors, confirming our results.^4,20,40^ The results from our multivariate analysis indicating a statistically significant association between the history of periodontitis and peri-implant diseases (14.825 OR [95%, CI 5.79% to 37.96%]) are comparable not only to those of Kordbacheh Changi et al,^21^ but also to other reported values in the literature.^9,13^

Despite the fact that the IDRA-tool considered prosthetic factors as one of the 8 risk vectors for peri-implant diseases and that other authors (e.g., Kordbacheh Changi et al^21^) highlighted poorly designed prosthesis reconstructions and cemented prostheses as major risk factors,^18^ the present analysis failed to show a statistically significant association between prosthetic reconstructions and occurrence of peri-implant diseases (OR = 0.835 [95% CI 34.9% to 19.99%], p = 0.6). This may be related to methodological aspects between the studies. An important advantage of the present study was the fact that all prosthetic restorations were performed by two experienced prosthodontists in cooperation with one single dental laboratory, thus limiting the number of operators, whereas in other studies, a larger number of dentists with various degrees of prosthetic experience and dental technicians completed the prosthetic rehabilitation.

The third highest statistically significant impact was the lack of keratinized tissue with an OR 13.914 (95% CI 4.96–38.99%).^29,36^ These results are also supported by other authors. For instance, Kungsadalpipob et al^22^ found a higher incidence of peri-implantitis at implants without keratinized mucosa (25%) vs those with keratinized mucosa (6.8%). Moreover, implants without keratinized tissue were associated with plaque accumulation, recession, an interproximal bone level loss above 3 mm, and peri-implantitis (p < 0.05). Rinke et al^31^ concluded that the presence of keratinized mucosa around implants was associated with a lower risk of peri-implantitis (OR 0.05 95%CI 0.01–0.25, p < 0.001). Smoking was also found to pose a statistically significant risk for developing peri-implantitis (OR 5.89, 95%CI 1.27–24.58, p = 0.0231). Additionally, when looking at subjects with implants ≥5 years in function (n = 169 implants), the same authors reported mucositis in 52% and PI in18% of the patients.

Interestingly, our multi-variate analysis revealed no statistically significant association between smoking and the occurrence of peri-implant diseases (Table 3). This may be related to the fact that we considered smokers as only those smoking ≥ 10 cigarettes/day, based on the fact that smoking ≥ 10 cigarettes/day is recognised as a grade modifier for periodontitis.^43^ In the literature, data related to smoking and occurrence of peri-implant diseases are contradictory. Some authors report a statistically significant association,^24^ whereas other studies are inconclusive.^25^ Moreover, the implant disease risk assessment tool did not include smoking as a vector for peri-implantitis risk.^14^

Contrary to data reported by other authors who emphasised a strong association between peri-implant diseases and uncontrolled diabetes or cardiovascular diseases,^42,44^ the present multivariate analysis found no statistically significant association between systemic diseases and peri-implant diseases (OR = 0.581, p > 0.05).

A limitation of the present study may be the fact that we considered only subjects who smoked ≥ 10 cigarettes/day to be smokers. This may have influenced the outcomes related to this risk indicator. Nonetheless, since smoking a higher number of cigarettes/day did not show any statistically significant association to peri-implant diseases, it is unlikely that a lower number of cigarettes/day might have indicated a positive association. A further limitation may be that we analysed the influence of all systemic diseases with no clear separation between disease groups, which could possibly possible explain our inability to show any statistically significant association between systemic diseases (e.g., diabetes, cardiovascular diseases) and peri-implant diseases as other authors did.

However, a strength of the present study is the fact that all included patients were patients of the same, single private dental clinic and all records related to their periodontal diagnoses, periodontal treatment and compliance with the maintenance periodontal therapy were available for analysis. A further advantage is the limited number (n = 2) of prosthodontists and having only one dental laboratory that performed the prosthetic rehabilitation, limiting thus the risk of bias for this factor.

Further prospective clinical trials are needed to clearly define the risk factors for developing peri-implant diseases using the current definitions for peri-implant diseases as indicated by EFP.

## CONCLUSION 

The present findings report a prevalence of peri-implant diseases (both on the subject and implant level) from a private practice setting comparable to data reported in the literature. Poor oral hygiene, a history of periodontitis, keratinized mucosa < 2 mm and a time in function ≥ 5 years have been associated with the occurrence of peri-implant diseases.
